# The Medical Lessons of the War

**Published:** 1915-02-06

**Authors:** 


### The Hospital

The Workers' Newspaper of Administrative Medicine and Institutional Life, Administration, National Insurance and Health.

No. 1494, Vol. LVII. SATURDAY, FEBRUARY 6, 1915 PRICE ONE PENNY.

### THE MEDICAL LESSONS OF THE WAR.

The news of the day which makes the first appeal to all of us is necessarily the activities and successes of our fighting forces. Everyone recognises—our foes now, as well as our friends—that the war has revealed no failure, either of military skill or of personal valour, in our sailors and soldiers, and the record of the past six months will take, most certainly, a proud place in the history of the British nation. Yet there are melancholy souls who seem to resign themselves to the constant contemplation of failures and shortcomings. By all means let us avoid the habit of self-delusion, but it is possible to practise this by blindness to good as well as by blindness to ill. The war has, without question, shown us some things which indicate national efficiency and justify national confidence, and of these we ought to cultivate a lively and sustained consciousness. It is in this spirit that we here refer to some of the medical aspects of the war.

First, on the question of preparation, there is abundant evidence that the present state of the medical services of the Navy and Army is no chance or haphazard development, but is the outcome of far-seeing judgment and valuable work. To give this statement a particular application we may make reference to the greatly improved standard of health in both of the fighting services, which, translated into action, has meant, and does mean, a practical increase of our combatant resources. Comparing the figures of to-day with those of thirty-five years ago, Sir Arthur May has recently shown that the "invaliding" rate has fallen from 35 to 15 per thousand, and the daily sick-rate from 47 to 25 per thousand. This latter improvement has an impressive quality when we learn that by it alone there are to-day 6,000 more sailors, or a number equal to the crews of three Dreadnoughts, fit for active service than would otherwise have been the case. For this result the nation has to thank the intelligent and sustained pressure exercised during years of peace by the Medical Department. Obstacles have been overcome, rebuffs have been ignored, and, if everything has not been gained, there is much to show that a steady determination to work for efficiency has characterised the medical authorities charged with the care of the health of our sailors. An improved standard of health has meant not only more men, but also better men, and these have been gained by work based on insight and pursued with courage.

Another ground on which may surely be found abundant reason for congratulation is the good health maintained by the soldiers fighting in France, and this the more when one realises the conditions to which they are exposed. A recent official figure gives the sick-rate of the British troops as 3 per cent., which is a lower figure than that of some of our home garrisons. So satisfactory a record is the product of many factors, among which medical efficiency must take a high place. This efficiency has been in operation long before the outbreak of the war, and has been made possible by a recognition—possibly a somewhat tardy recognition—by the military authorities of the truth that a healthy army means a fighting machine increased both in numbers and in quality. The teaching mission of the doctors has spread downwards as well as upwards, and has taught the private that it is his interest as well as his duty to conform to directions intended to promote bodily vigour as well as to prevent disease. Hence the figures above quoted, and in despite of conditions which, to quote a standard military authority, would in old days have caused an army to melt rapidly away. We have not the least desire to paint everything *couleur de rose*, and we have no doubt that many improvements are yet possible, but in recognising this possibility let us not forget legitimate triumphs and the sound work of which they are the expression. Of these the medical profession may boast a considerable share; but there are abundant evidences that all the departments concerned have loyally devoted themselves to anticipate and to prepare the supplies which are essential to the welfare of the individual soldier considered as a fighting unit in an army on active service.

A special preventive measure is, of course, inoculation against typhoid. We are not going now to argue the value of this measure, but even those who criticise the method must allow that it is the outcome of much patient and ingenious research, and that it holds a high place in the judgment of many competent scientific minds. The credit of this work belongs to British pathologists and clinicians.

It is only possible in general terms to refer to the arrangements for the care of the wounded, which all authorities agree have never been so well organised or so well conducted. The transport of injured men from the trenches to the hospitals, the qualifications of the surgical staff, and the equipment of the hospitals have all alike received the endorsement of the most trustworthy witnesses. The voice of criticism in reference to details is not silent, and no sensible person would wish it to be silent. But let also the voice of appreciation be heard, and when our shortcomings are recited, let us recall our successes and in view of these be of good courage.

